# Supplementary material for: Leukocyte Telomere Length as a Marker of Chronic Complications in Type 2 Diabetes Patients: A Risk Assessment Study
Source: Int J Mol Sci. 2024 Dec 31;26(1):290. doi: 10.3390/ijms26010290 (PMC11719939; doi:10.3390/ijms26010290)
Supplement: Supplementary file 1 [file ijms-26-00290-s001.zip › Table S1. Caucasian patients with diabetic polyneuropathy demographics and their clinical and biochemical characteristics broken down into tertiles according to leukocyte telo.pdf]

Table S1. Caucasian patients with diabetic polyneuropathy demographics and their clinical and biochemical characteristics broken down into tertiles according to leukocyte telomere length.

Parameters	Total	LTL tertile			*P* value	Test
		tertile 1	tertile 2	tertile 3		
n	109	38	35	36		
LTL	1.06 (0.40)	0.66 (0.17)	1.04 (0.08)	1.49 (0.31)		
Men [%]	53.20	50.00	54.30	55.60	0.881	χ^2
Age [years]	65.73 (9.76)	66.79 (9.94)	64.37 (9.27)	65.94 (10.14)	0.569	*ANOVA*
Duration of diabetes [years]	16.00 (12.00)	17.00 (15.00)	15.50 (10.00)	17.00 (12.00)	0.387	KW
Age at diagnosis [years]	48.95 (10.46)	49.05 (11.16)	48.88 (9.83)	48.91 (10.56)	0.996	*ANOVA*
BMI [kg/m^2]	34.31 (6.66)	34.17 (6.36)	33.67 (6.24)	35.07 (7.44)	0.704	*ANOVA*
WC [cm]	114.67 (14.30)	116.08 (13.40)	112.21 (13.42)	115.61 (16.01)	0.464	*ANOVA*
HbA1c [%]	8.20 (2.20)	8.20 (1.90)	8.00 (3.50)	7.80 (2.25)	0.727	KW
CRP [mg/l]	4.57 (10.31)	4.37 (10.00)	5.50 (10.60)	4.09 (18.22)	0.948	KW
vitamin D_3 [ng/ml]	22.43 (10.57)	20.77 (9.59)	24.46 (10.39)	22.21 (11.65)	0.328	*ANOVA*
TC [mg/dl]	151.22 (60.54)	164.95 (77.99)	153.43 (49.79)	134.58 (44.47)	0.096	*ANOVA*
LDL [mg/dl]	75.28 (40.99)	80.78 (44,32)	78.17 (41.75)	66.81 (36.18)	0.362	*ANOVA*
HDL [mg/dl]	36.12 (10.29)	37.79 (11.64)	36.51 (8.70)	33.97 (10.11)	0.225	*ANOVA*
Non-HDL [mg/dl]	115.65 (57.58)	128.61 (75.52)	117.00 (46.63)	100.67 (40.93)	0.115	*ANOVA*
TG [mg/dl]	143.00 (115.00)	141.00 (119.00)	152.00 (118.00)	134.50 (120.00)	0.574	KW
eGFR [<60 ml/min/1,73 m^2] [%]	33.90	31.60	31.40	38.90	0.746	χ^2
DPI [VAS]	7.00 (4.00)	8.00 (4.00)	6.00 (8.00)	7. (3.50)	0.147	KW

Data are expressed as mean (SD), median (interquartile range) or no. (percentage) for categorical variables. *P*-values compare LTL 3rd tertile (max) to LTL 1st tertile (min). Statistically significant *P* -values bolded. LTL - leukocyte telomere length, χ^2 - Chi-square test, ANOVA – one-way ANOVA test, KW- Kruskal-Wallis test, SD – standard deviation, BMI – body mass index, WC – waist circumference, HbA1c – hemoglobin A1C (glycated hemoglobin), CRP – C-reactive protein, TC – total cholesterol, LDL – low-density lipoprotein, HDL – high-density lipoprotein, Non HDL – non-high-density lipoprotein, TG – triglycerides, eGFR – estimated glomerular filtration rate, DPI – determination of pain intensity, VAS - visual analogue scale.
