# Supplementary material for: Leukocyte Telomere Length as a Marker of Chronic Complications in Type 2 Diabetes Patients: A Risk Assessment Study
Source: Int J Mol Sci. 2024 Dec 31;26(1):290. doi: 10.3390/ijms26010290 (PMC11719939; doi:10.3390/ijms26010290)
Supplement: Supplementary file 1 [file ijms-26-00290-s001.zip › Table S2. Caucasian patients with diabetic foot ulcer demographics and their clinical and biochemical characteristics broken down into tertiles according to leukocyte telomere length..pdf]

Table S2. Caucasian patients with diabetic foot ulcer demographics and their clinical and biochemical characteristics broken down into tertiles according to leukocyte telomere length.

Parameters	Total	LTL tertile			*P* value	Test
		tertile 1	tertile 2	tertile 3		
n	43	14	12	17		
LTL	1.04 (0.35)	0.64 (0.17)	1.05 (0.06)	1.38 (0.20)		
Men [%]	74.40	64.30	91.70	70.60	0.251	χ^2
Age [years]	63.12 (8.67)	63.36 (9.38)	60.42 (7.88)	64.82 (8.65)	0.410	*ANOVA*
Duration of diabetes [years]	15.00 (10.00)	15.00 (15.00)	15.50 (10.00)	15.00 (10.00)	0.832	KW
Age at diagnosis [years]	46.77 (9.77)	47.00 (11.57)	45.25 (9.30)	47.65 (8.92)	0.812	*ANOVA*
BMI [kg/m^2]	32.15 (5.80)	33.20 (6.83)	32.47 (4.91)	31.07 (5.61)	0.594	*ANOVA*
WC [cm]	111.89 (14.20)	114.71 (15.78)	114.02 (9.28)	108.06 (15.56)	0.366	*ANOVA*
HbA1c [%]	7.80 (2.40)	8.30 (1.50)	8.05 (2.35)	7.50 (2.40)	0.770	KW
CRP [mg/l]	11.46 (30.66)	8.48 (14.57)	17.46 (37.40)	17.53 (32.90)	0.456	KW
vitamin D$_3$ [ng/ml]	24.34 (12.39)	21.59 (12.14)	23.46 (9.36)	27.24 (14.39)	0.469	*ANOVA*
TC [mg/dl]	135.28 (40.11)	143.29 (43.50)	139.42 (45.02)	125.76 (33.58)	0.442	*ANOVA*
LDL [mg/dl]	67.52 (29.47)	69.08 (17.66)	76.17 (37.46)	60.24 (30.20)	0.304	*ANOVA*
HDL [mg/dl]	32.88 (9.59)	33.50 (6.19)	32.17 (9.27)	32.88 (12.27)	0.864	*ANOVA*
Non-HDL [mg/dl]	102.74 (37.40)	109.93 (41.09)	108.17 (39.11)	93.00 (32.86)	0.365	*ANOVA*
TG [mg/dl]	138.00 (98.00)	140.50 (63.00)	150.50 (95.00)	137.00 (117.00)	0.893	KW
eGFR [<60 ml/min/1,73 m^2] [%]	41.90	42.90	33.30	47.10	0.758	χ^2
DPI [VAS]	6.00 (8.00)	5.00 (8.00)	6.50 (6.00)	6.50 (5.50)	0.893	KW

Data are expressed as mean (SD), median (interquartile range) or no. (percentage) for categorical variables. *P*-values compare LTL 3rd tertile (max) to LTL 1st tertile (min). Statistically significant *P* -values bolded. LTL - leukocyte telomere length, χ^2 - Chi-square test, ANOVA – one-way ANOVA test, KW- Kruskal-Wallis test, SD – standard deviation, BMI – body mass index, WC – waist circumference, HbA1c – hemoglobin A1C (glycated hemoglobin), CRP – C-reactive protein, TC – total cholesterol, LDL – low-density lipoprotein, HDL – high-density lipoprotein, Non HDL – non-high-density lipoprotein, TG – triglycerides, eGFR – estimated glomerular filtration rate, DPI – determination of pain intensity, VAS - visual analogue scale.
