# Supplementary material for: Leukocyte Telomere Length as a Marker of Chronic Complications in Type 2 Diabetes Patients: A Risk Assessment Study
Source: Int J Mol Sci. 2024 Dec 31;26(1):290. doi: 10.3390/ijms26010290 (PMC11719939; doi:10.3390/ijms26010290)
Supplement: Supplementary file 1 [file ijms-26-00290-s001.zip › Table S3. Logistic regression risk assessment for testing the association of LTL 3rd tertile with selected variables.pdf]

Table S3. Logistic regression risk assessment for testing the association of LTL 3rd tertile with selected variables

Model description	Variables	OR	95 % CI		*P* value
Crude model	Age at diagnosis	1.01	1.00	1.02	0.228
	BMI	1.10	0.60	1.99	0.763
	WC	1.00	0.97	1.02	0.863
	Vitamin D_3	1.12	0.82	1.52	0.490
	TC	0.74	0.60	0.92	**0.006**
	LDL	0.83	0.69	1.00	**0.047**
	HDL	0.35	0.10	1.17	0.088
	Non-HDL	0.75	0.62	0.92	**0.005**
	DN	1.31	0.63	2.73	0.472
	DPN	0.95	0.42	2.11	0.891
	DFU	1.45	0.69	3.04	0.325
	DR	1.92	0.90	4.12	0.092
Adjusted for sex and age model	Age at diagnosis	1.01	0.99	1.02	0.382
	BMI	1.17	0.63	2.15	0.623
	WC	1.00	0.97	1.02	0.862
	Vitamin D_3	1.11	0.81	1.51	0.533
	TC	0.71	0.56	0.91	**0.006**
	LDL	0.83	0.67	1.01	0.061
	HDL	0.37	0.10	1.31	0.122
	Non-HDL	0.73	0.58	0.91	**0.006**
	DN	1.24	0.58	2.68	0.577
	DPN	0.88	0.39	2.01	0.769
	DFU	1.46	0.67	3.15	0.338
	DR	1.87	0.87	4.02	0.111

Statistically significant *P* -values bolded. LTL - leukocyte telomere length, OR: Odds ratio, CI: Confidence interval, BMI – body mass index, WC – waist circumference, TC – total cholesterol, LDL – low-density lipoprotein, HDL – high-density lipoprotein, Non HDL – non-high-density lipoprotein, DN – diabetic nephropathy, DPN – diabetic polyneuropathy, DFU – diabetic foot ulcer, DR – diabetic retinopathy.
